# Proximity Utilizing Biotinylation of Nuclear Proteins *in vivo*

**DOI:** 10.5195/cajgh.2014.165

**Published:** 2015-06-15

**Authors:** Arman Kulyyassov, Gulsamal Zhubanova, Erlan Ramanculov, Vasily Ogryzko

**Affiliations:** 1 National Center for Biotechnology, Astana, Kazakhstan; 2 Institut Gustave Roussy, Villejuif, France

**Keywords:** protein-protein interaction, proximity, biotinylation

## Abstract

**Introduction:**

The human genome consists of roughly 30,000 genes coding for over 500,000 different proteins, of which more than 10,000 proteins can be produced by the cell at any given time (the cellular “proteome”). It has been estimated that over 80% of proteins do not operate alone, but in complexes. These protein-protein interactions (PPI) are regulated by several mechanisms. For example, post-translational modifications (methylation, acetylation, phosphorylation, or ubiquitination) or metal-binding can lead to conformational changes that alter the affinity and kinetic parameters of the interaction. Many PPIs are part of larger cellular networks of interactions or interactomes. Indeed, these interactions are at the core of the entire interactomics system of any living cell, and so, aberrant PPIs are the basis of multiple diseases, such as neurodegenerative diseases and cancer. The objective of this study was to develop a method of monitoring protein-protein interactions and proximity dependence in vivo.

**Methods:**

The biotin ligase BirA was fused to the protein of interest, and the Biotin Acceptor Peptide (BAP) was fused to an interacting partner to make the detection of its biotinylation possible by western blot or mass spectrometry.

**Results:**

Using several experimental systems (BirA.A + BAP.B), we showed that the biotinylation is interaction/proximity dependent. Here, A and B are the next nuclear proteins used in the experiments – 3 paralogues of heterochromatin protein HP1α (CBX5), HP1β (CBX1), HP1γ (CBX3), wild type and transcription mutant factor Kap1, translesion DNA polymerase PolH and E3, ubiquitin ligase RAD18, Proliferative Cell Nuclear Antigen (PCNA), ubiquitin Ub, SUMO-2/3, different types and isoforms of histones H2A, H2Az, H3.1, H3.3, CenpA, H2A.BBD, and macroH2A. The variant of this approach is termed PUB-NChIP (Proximity Utilizing Biotinylation with Native Chromatin Immuno-precipitation) and is designed to purify and study the protein composition of chromatin in proximity to the nuclear protein of interest. Using the RAD18 protein as a model, we demonstrated that the RAD18-proximal chromatin is enriched in some H4 acetylated species. Moreover, the RAD18-proximal chromatin containing a replacement histone H2Az has a different pattern of H4 acetylation.

**Conclusion:**

Progress in the last decade in cancer drug therapy has led us to the conclusion that the nucleus of eukaryotic cells is an active site for many cellular processes important to the development of cancer. These processes include changes in genetic and epigenetic landscape (e. g. methylation of DNA, modification of histones) and the expression levels of transcription factors, which regulates gene products (e.g. hypoxia-inducible factor 1α (HIF-1α) in chronic anemia, etc.) where protein-protein interactions play important role. Understanding the nature of protein-protein interactions may improve design strategies for small-molecule PPI modulators. PPI assay technologies that closely reflect physiological conditions hold the key to developing specific anti-cancer drugs.

